# Supplementary figures and images for: Comparative Application of BioID and TurboID for Protein-Proximity Biotinylation
Source: Cells. 2020 Apr 25;9(5):1070. doi: 10.3390/cells9051070 (PMC7290721; doi:10.3390/cells9051070)

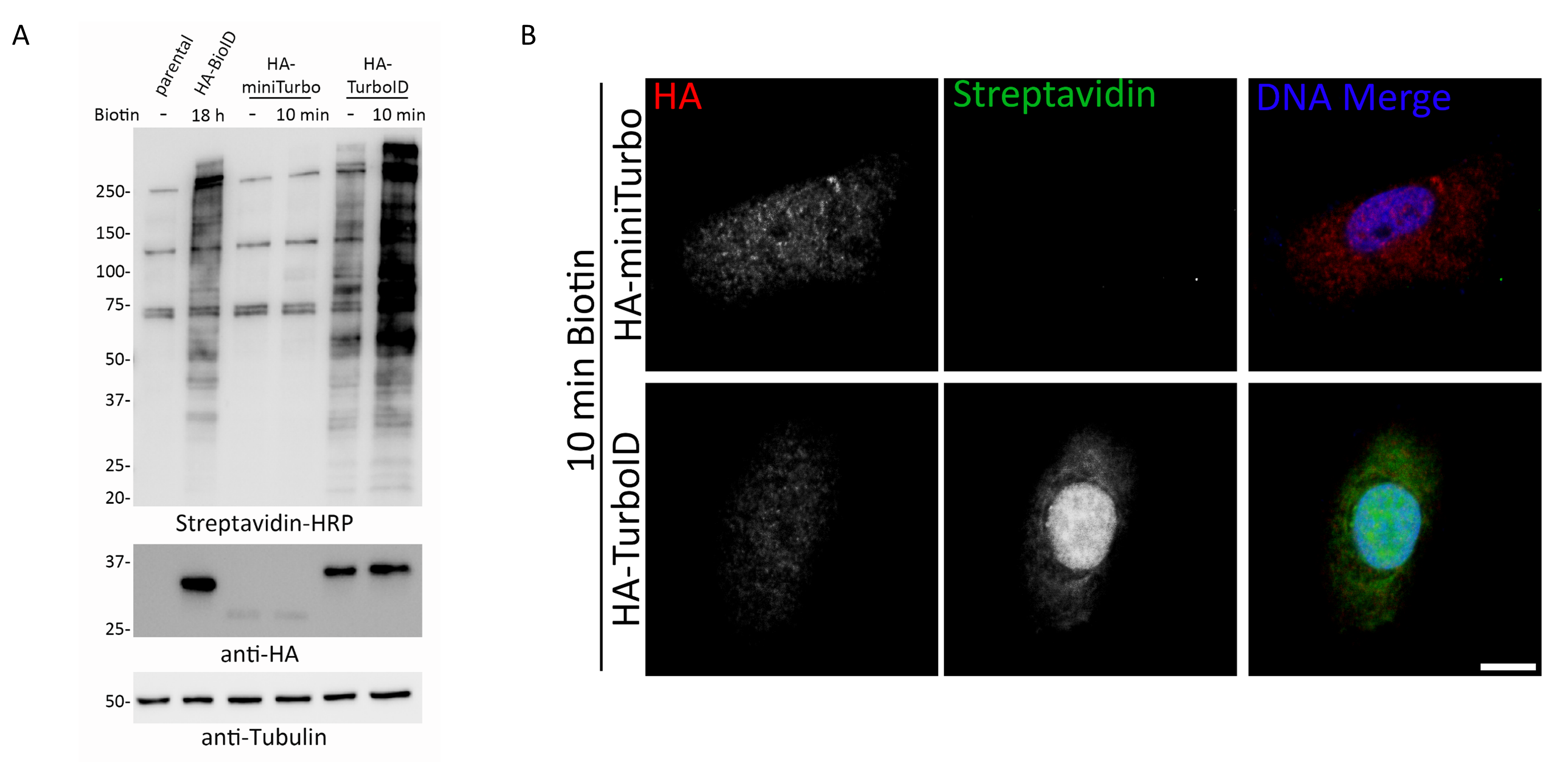

Supplement: Supplementary file 1 [file cells-09-01070-s001.zip › Figure S1.tif]

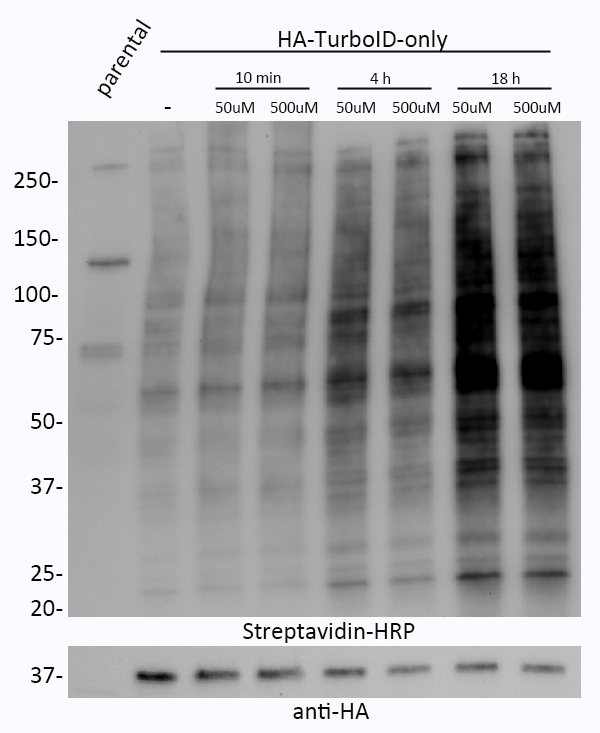

Supplement: Supplementary file 1 [file cells-09-01070-s001.zip › Figure S2-Biotin Concentration Timecourse.tif]

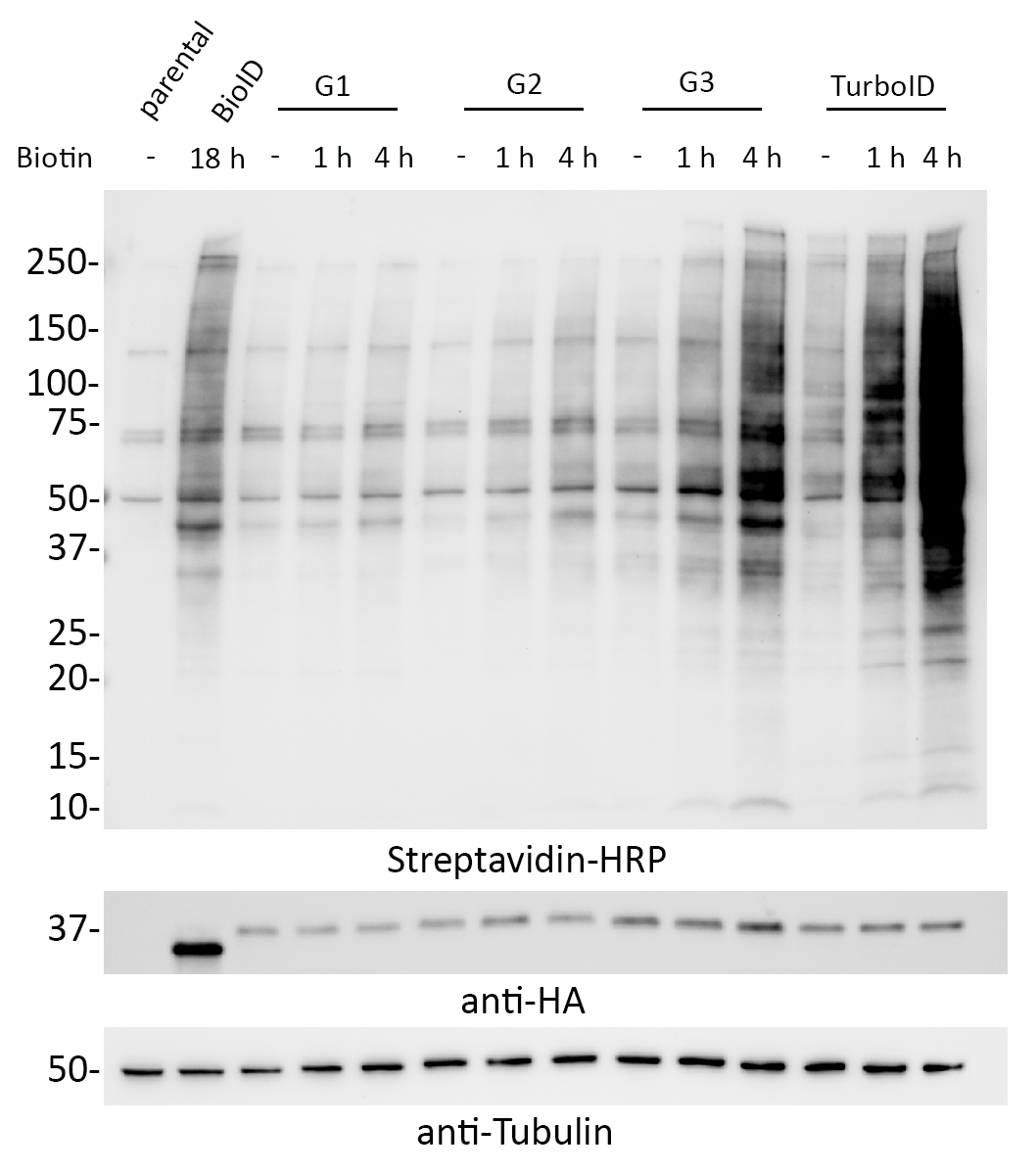

Supplement: Supplementary file 1 [file cells-09-01070-s001.zip › Figure S3 - TurboGs Figure.tif]

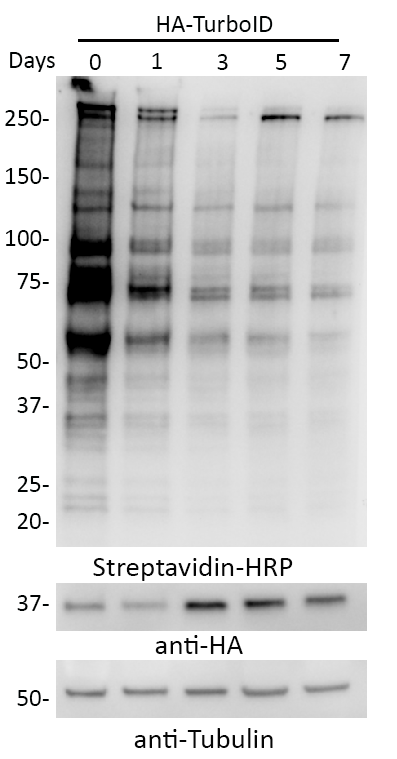

Supplement: Supplementary file 1 [file cells-09-01070-s001.zip › Figure S4 - TurboID Dialyzed Serum Timecourse Figure.tif]

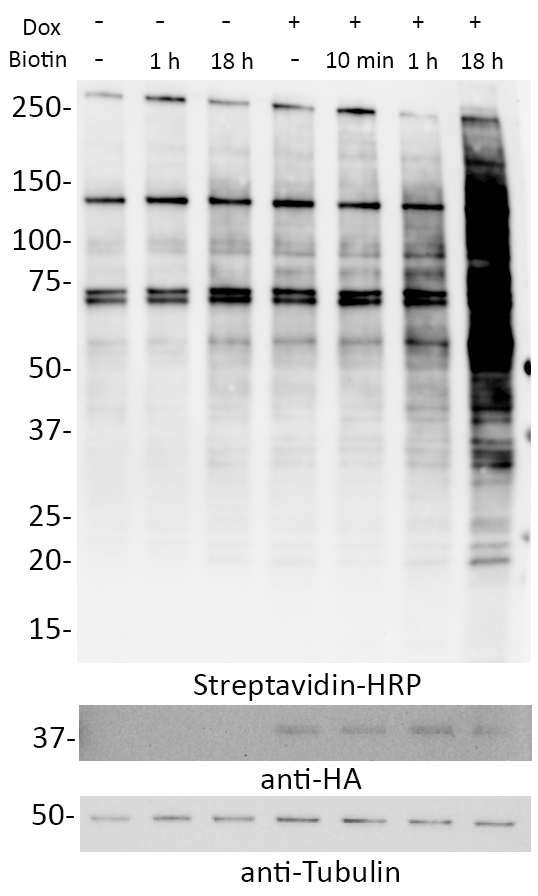

Supplement: Supplementary file 1 [file cells-09-01070-s001.zip › Figure S5 - pRetroX.tif]

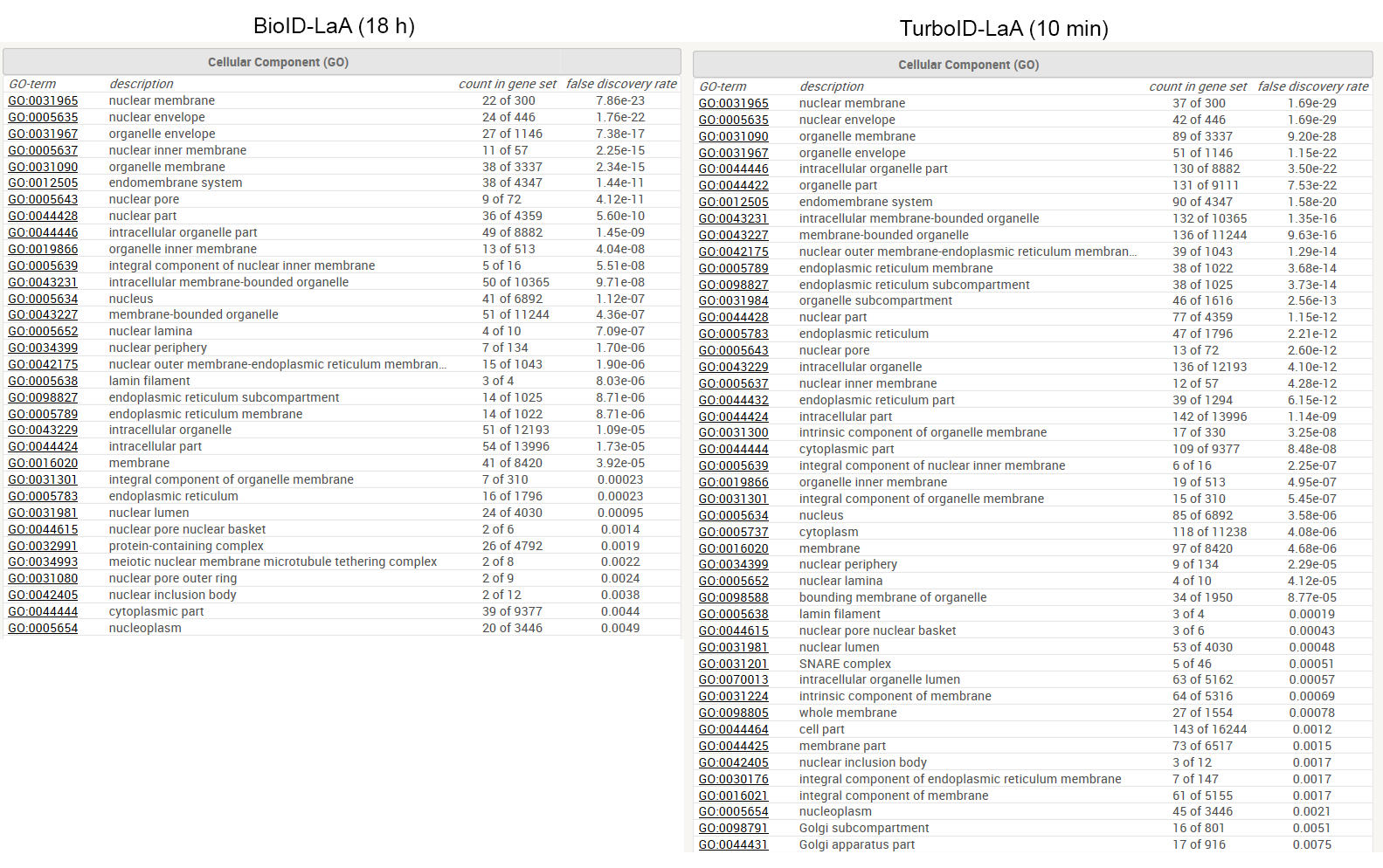

Supplement: Supplementary file 1 [file cells-09-01070-s001.zip › Table S2.tif]

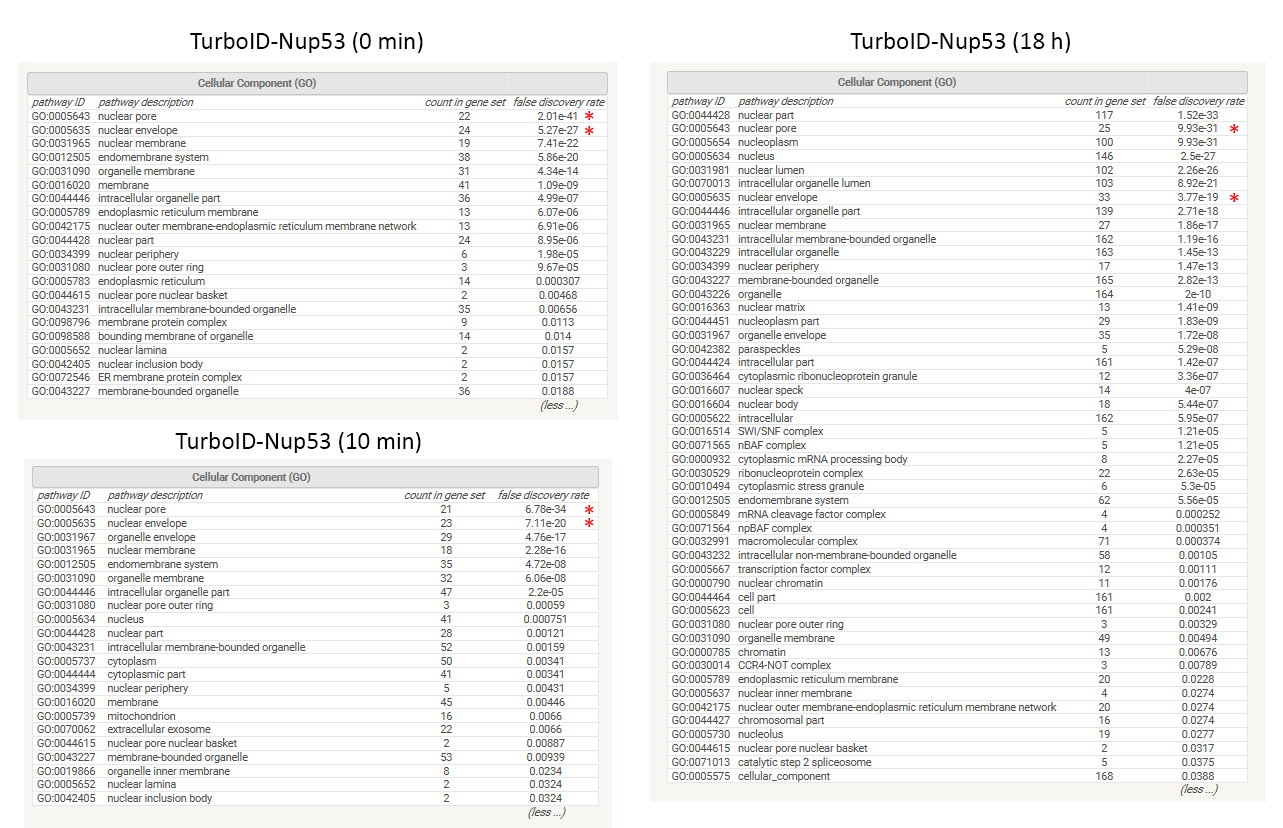

Supplement: Supplementary file 1 [file cells-09-01070-s001.zip › Table S6.tif]

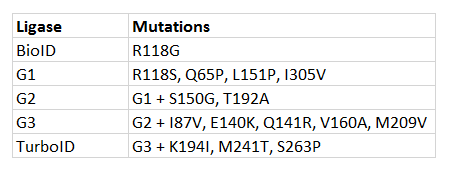

Supplement: Supplementary file 1 [file cells-09-01070-s001.zip › Table S7.tif]
